# Homeobox gene Dlx-2 is implicated in metabolic stress-induced necrosis

**DOI:** 10.1186/1476-4598-10-113

**Published:** 2011-09-14

**Authors:** Su Yeon Lee, Hyun Min Jeon, Cho Hee Kim, Min Kyung Ju, Hye Sun Bae, Hye Gyeong Park, Sung-Chul Lim, Song Iy Han, Ho Sung Kang

**Affiliations:** 1 Department of Molecular Biology, College of Natural Sciences, Pusan National University, Pusan 609-735, Korea; 2 Nanobiotechnology Center, Pusan National University, Pusan 609-735, Korea; 3 Research Center for Resistant Cells, College of Medicine, Chosun University, Gwangju 501-759, Korea; 4 Department of Pathology, College of Medicine, Chosun University, Gwangju 501-759, Korea; 5 DNA Identification Center, National Forensic Service, Seoul 158-707, Korea

## Abstract

**Background:**

In contrast to tumor-suppressive apoptosis and autophagic cell death, necrosis promotes tumor progression by releasing the pro-inflammatory and tumor-promoting cytokine high mobility group box 1 (HMGB1), and its presence in tumor patients is associated with poor prognosis. Thus, necrosis has important clinical implications in tumor development; however, its molecular mechanism remains poorly understood.

**Results:**

In the present study, we show that Distal-less 2 (Dlx-2), a homeobox gene of the Dlx family that is involved in embryonic development, is induced in cancer cell lines dependently of reactive oxygen species (ROS) in response to glucose deprivation (GD), one of the metabolic stresses occurring in solid tumors. Increased Dlx-2 expression was also detected in the inner regions, which experience metabolic stress, of human tumors and of a multicellular tumor spheroid, an *in vitro *model of solid tumors. Dlx-2 short hairpin RNA (shRNA) inhibited metabolic stress-induced increase in propidium iodide-positive cell population and HMGB1 and lactate dehydrogenase (LDH) release, indicating the important role(s) of Dlx-2 in metabolic stress-induced necrosis. Dlx-2 shRNA appeared to exert its anti-necrotic effects by preventing metabolic stress-induced increases in mitochondrial ROS, which are responsible for triggering necrosis.

**Conclusions:**

These results suggest that Dlx-2 may be involved in tumor progression via the regulation of metabolic stress-induced necrosis.

## Background

Rapidly growing malignant tumors experience hypoxia and nutrient (e.g., glucose) deprivation, which occurs because of insufficient blood supply. Most tumor cells display higher rates of aerobic glycolysis; thus, glucose deprivation (GD) may be exacerbated in the inner regions of solid tumors. Under circumstances of hypoxia and nutrient deprivation, tumor cells either survive by overcoming the cytotoxic effects of such metabolic stresses via the activation of certain signal transduction pathways and gene regulatory mechanisms, or undergo cell death, especially in the innermost regions [1-4]. In tumors, metabolic stress-induced cell death mostly occurs by necrosis because most apoptotic and/or autophagic programs are limited during the development of human tumors [5]. In fact, necrosis is commonly found in the core region of solid tumors. In apoptosis, cells are progressively fragmented into apoptotic bodies that are removed by professional phagocytic cells and in autophagic cell death, autophagosomes break down the damaged subcellular organelles. However, necrosis is characterized by the rupture of the cell membrane and release of cellular contents, including high mobility group box 1 (HMGB1) into the extracellular microenvironment, thereby causing a massive inflammatory response [6-10]. Necrotic cells recruit immune inflammatory cells, which exert a tumor-promoting activity by inducing angiogenesis, cancer cell proliferation, and invasiveness [9,10]. The HMGB1 protein is a highly conserved nuclear protein, which acts as a transcriptional regulator by controlling the activities of many transcription factors, including p53 and the Rel/NF-κB family [11-13]. Within the cytosol, HMGB1 also induces autophagy by interacting with and regulating Beclin 1 as a cofactor [14]. In addition, HMGB1 is released from necrotic cells and secreted by activated macrophages and functions as an extracellular signaling molecule [11-13]. HMGB1 binds to several receptors, including the receptor for advanced glycation end products (RAGEs) and Toll-like receptors (TLR)-2 and TLR-4, and promotes inflammation, cell proliferation, and tumor growth and metastasis. Thus, HMGB1 acts as a pro-inflammatory and tumor-promoting cytokine when released into the extracellular space during necrosis [11-13]. Recently, HMGB1 is known to be also released during autophagy and late apoptosis; for instance, anticancer agents that enhance autophagy and apoptosis cause HMGB1 release in cancer cells. In addition, the HMGB1 protein triggers autophagy or apoptosis in cancer cells, depending on its redox status. Reduced HMGB1 induces Beclin1-dependent autophagy and promotes tumor resistance to several chemotherapeutic agents, whereas oxidized HMGB1 induces apoptosis via the mitochondrial caspase-9/-3 pathway [15]. In cancer, overexpression of HMGB1 is associated with the hallmarks of cancer, including sustained proliferative potential and replicative immortality, angiogenesis, apoptosis resistance, self-sufficient growth, insensitivity to suppressors of growth, inflammation, and invasion and metastasis [16]. Thus, necrosis has the tumor-promoting potential as "a reparative cell death." Development of a necrotic core in cancer patients is correlated with increased tumor size, high-grade disease, and poor prognosis, such as emergence of chemoresistance and metastases [1-3]. Thus, metabolic stress-induced necrosis has important clinical implications. However, in contrast to programmed cell death, apoptosis, and autophagic cell death, the molecular mechanism underlying metabolic stress-induced necrosis in tumors is less investigated because it is generally considered as an accidental and genetically unprogrammed form of cell death.

The Distal-less (Dlx) homeobox gene family, a homolog of Drosophila distal-less (Dll), is crucial for embryonic development, morphogenesis, and tissue homeostasis, including neurogenesis and the formation of the distal regions of extending appendages in invertebrates and vertebrates [17,18]. Homeobox genes are characterized by a highly conserved 60-amino acid homeodomain, which binds DNA elements containing a TAAT core motif. In humans, there are 6 Dlx genes, which exist as 3 bigene clusters: Dlx-1/Dlx-2, Dlx-5/Dlx-6, and Dlx-3/Dlx-7 [19]. A growing number of homeobox genes have been shown to be deregulated in a variety of human tumors, and their deregulation is known to enhance cell survival and proliferation and prevent differentiation [20-23]. Deregulation of Dlx gene expression has also been reported in many human solid tumors and hematologic malignancies. For instance, Dlx-4 overexpression is observed in ovarian and breast cancers and strongly correlates with high tumor grade, advanced disease stage, and poor prognosis [24,25]. In addition, Dlx-5 is also unregulated in several human solid tumors, including lung, breast, and ovarian cancers, and T-cell lymphomas, and contributes to tumor progression [26-29]. Dlx-5 has been shown to promote tumor cell proliferation by directly binding the MYC promoter and upregulating MYC [30]. Dlx-7 is also known to regulate MYC expression in erythroleukemia cells [31].

In this study, we tried to identify the molecules that are involved in necrosis. Previously, we demonstrated that GD, one of the stresses that cause metabolic stress in tumors, could induce necrosis and HMGB1 release into the extracellular space in A549, HepG2, and MDA-MB-231 cells [32]. We also showed that phorbol-12-myristate-13-acetate (PMA), a protein kinase C (PKC) activator, prevented GD-induced necrosis in A549 cells, and switched the cell death mode to apoptosis by inhibiting reactive oxygen species (ROS) production by regulating manganese superoxide dismutase (MnSOD) and copper-zinc SOD [32]. In this study, cDNA microarray analysis revealed that a homeobox gene Dlx-2 is induced in A549 cells that undergo necrosis but not in those that die by apoptosis. We also demonstrate that Dlx-2 is implicated in metabolic stress-induced necrosis, suggesting a possible role(s) of Dlx-2 in tumor progression.

## Results

### Dlx-2 is induced dependently of ROS during metabolic stress-induced necrosis

**Figure 1:**
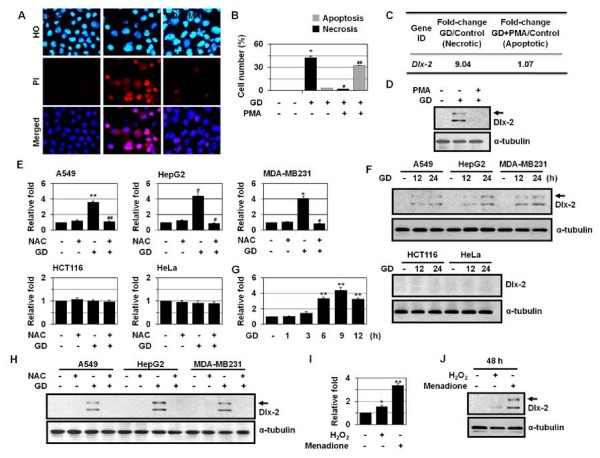
**Induction of Dlx-2 during metabolic stress-induced necrosis**. (A, B) A549 cells were pretreated with PMA and treated with GD for 18 h, and then stained with HO/PI (A), and apoptotic and necrotic cells were scored (B). (C, D) A549 cells were pretreated with PMA and treated with GD for 12 h and microarray analysis was performed. The numbers mean fold increase in expression as compared with GD-untreated control cells (C). The cells were analyzed using Western blotting (D). (E) Different types of cells were pretreated with NAC and treated with GD for 12 h, and then analyzed by real-time PCR for Dlx-2 expression. (F) Cells were treated with GD and then analyzed using Western blotting. (G) MDA-MB-231 cells were treated with GD, and then analyzed by real-time PCR for Dlx-2 expression. (H) Cells were pretreated with NAC and treated with GD for 12 h, and then analyzed using Western blotting. (I, J) MCF-7 cells were treated with H_2_O_2 _and menadione for 48 h, and then analyzed by real-time PCR (I) and Western blotting for Dlx-2 expression (J). The values obtained from HO/PI staining and real-time PCR are expressed as mean ± SE (n = 3). *P < 0.05, **P < 0.01 versus control; ^#^*P *< 0.05, ^##^*P *< 0.01 versus GD-treated cells. Arrow shown in panels D, F, H, and J, a putative modified form of Dlx-2.

The aim of this study was to identify the molecules that are involved in metabolic stress-induced necrosis. As demonstrated previously [32], PMA, a PKC activator, prevented GD-induced necrosis in A549 cells and switched the cell death mode to apoptosis (Figure 1A and 1B). To identify necrosis-linked molecules, we analyzed the gene expression profiling of A549 cells that were treated with GD or GD+PMA by cDNA microarrays. Of the 3,096 genes analyzed, approximately 200 were upregulated > 2-fold and approximately 150 were downregulated > 2-fold (GEO accession no. GSE24271). One of the GD-inducible genes was Dlx-2, a homeobox gene of the Dlx family (Figure 1C); the Dlx-2 level was increased 9.04-fold during necrosis, whereas its level was not changed during apoptosis. Western blot analysis confirmed GD induction of Dlx-2 in GD-treated A549 cells but not in A549 cells that were pretreated to PMA and then treated with GD, indicating that Dlx-2 expression increases only in the presence of necrosis (Figure 1D).

Dlx-2 has been shown to be expressed at higher levels in human breast cancers compared to other Dlx genes, including Dlx-1, Dlx-3, Dlx-4, and Dlx-6, although its precise roles in tumor biology are not clear [29]. Therefore, we investigated whether Dlx-2 is involved in metabolic stress-induced necrosis. In two-dimensional cultures, GD can induce either apoptosis or necrosis depending on the cell types; it induces necrosis in A549, HepG2, and MDA-MB-231 cells, while it induces apoptosis in HeLa and HCT116 cells [32,33]. Thus, we examined the expression of Dlx-2 in the cancer cell lines that undergo either necrosis or apoptosis upon GD treatment. Real-time quantitative PCR showed induction of Dlx-2 by GD in A549 (3.62-fold), HepG2 (4.41-fold), and MDA-MB-231 cells (4.07-fold), but not in HeLa and HCT116 cells (Figure 1E). Western blot analysis confirmed GD induction of Dlx-2 in A549, HepG2, and MDA-MB-231 cells but not in HeLa and HCT116 cells (Figure 1F). The induction of Dlx-2 was apparent at 6 h of GD, before the time point when necrosis was observed (Figure 1G). We observed that anti-Dlx-2 antibody recognized 2 bands (40 kDa and 34 kDa) (Additional file 1. Figure S1). Both bands were increased in response to GD treatment. The lower band seems to be Dlx-2. Dlx-2 is known to be modified by phosphorylation, and therefore, the higher band is thought to be a post-transcriptionally modified form of Dlx-2. To confirm the possible modification of Dlx-2, we transfected the Dlx-2 expression vector. Dlx-2 overexpression caused morphological changes typical of cells with a mesenchymal phenotype in MCF-7 cells (Additional file 1. Figure S1), indicating that Dlx-2 may trigger the epithelial to mesenchymal transition that plays a key role(s) in embryonic development, wound healing, and cancer metastasis. 2 bands were recognized by anti-Dlx-2 antibody (Additional file 1. Figure S1). It remains to be elucidated whether the upper band is really a modified Dlx-2 and what mechanism is responsible for the modification and what is the biological relevance of the modification in metabolic stress-induced necrosis.

Because mitochondrial ROS especially O_2_^- ^have been shown to mediate GD-induced cytotoxicity and necrotic cell death [34-36], we examined whether the Dlx-2 induction is linked to ROS. Dlx-2 induction by GD was inhibited by treatment with the antioxidant *N*-acetylcysteine (NAC) in A549, HepG2, and MDA-MB-231 cells (Figure 1E and 1H). To confirm ROS-dependent Dlx-2 induction, we treated MCF-7 cells with H_2_O_2 _and menadione (a O_2_^- ^generator). Increased Dlx-2 mRNA and protein expression was observed by real-time PCR and Western blotting, respectively (Figure 1I and 1J). O_2_^- ^was a more potent inducer of Dlx-2 than H_2_O_2_. Similar results were obtained with MDA-MB-231 cells (data not shown). These results demonstrate the redox-sensitivity of Dlx-2 expression.

### Dlx-2 is induced in multicellular tumor spheroids

**Figure 2:**
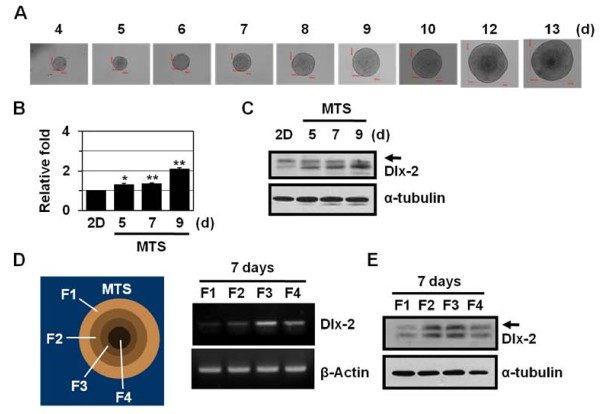
**Dlx-2 shRNA prevents metabolic stress-induced necrosis in MTS**. (A) Formation, growth, and morphology of MTSs derived from MCF-7 cells, which were cultured for up to 13 days. (B-C) MCF-7 spheroids cultured on agarose for the indicated times were analyzed by real-time PCR for Dlx-2 expression (B). The values are expressed as mean ± SE (n = 3). *P < 0.05, **P < 0.01 versus two-dimensional cultured cells. The MCF-7 spheroids were also analyzed using Western blotting with antibodies against Dlx-2 and α-tubulin (C). (D-E) After 7 days of MCF-7 MTS culture, the MTSs were dissociated into subpopulations of cells from different locations in the spheroids, as described in Materials and Methods. The cells isolated from different locations within the MCF-7 spheroids were analyzed by RT-PCR using primers for Dlx-2 and β-actin (D). The cells were also analyzed using Western blotting with antibodies against Dlx-2 and α-tubulin (E). Arrow in panels C and E, a putative modified form of Dlx-2.

We examined Dlx-2 protein expression using three-dimensional multicellular tumor spheroids (MTSs). MTSs closely mimic many characteristics of poorly vascularized solid tumors, including tumor growth pattern and necrotic core formation, and hence are used for *in vitro *models of solid tumors. As the MTSs mature, a proliferation gradient is observed, with proliferating cells at the periphery, cell-cycle arrested cells in the inner regions, and necrotizing cells in the core regions [37,38]. The innermost cells die by necrosis due to insufficient supply of oxygen and glucose [37,38]. As demonstrated previously [33,39], MCF-7 cells form tightly packed, rounded spheroids of homogeneous size (Figure 2A). We found increased expression of Dlx-2 with extended MTS culture (Figure 2B and 2C): 2.10-fold (*P *= 0.002) Dlx-2 induction was observed in 9-day MTSs, which may experience metabolic stress. To determine the expression of Dlx-2 in MTSs, the spheroids were selectively dissociated to yield cells from 4 discrete regions within the spheroid. Dlx-2 was detected in the inner F2 and F3 fractions and the innermost F4 fraction but not the outermost F1 fraction (Figure 2D and 2E). p27Kip1, a CKI family protein that is involved in cell cycle arrest, is regulated by HIF-1α, and its increased expression has been detected in the F2 and F3 fractions [40]; hence, the F2 and F3 fractions are thought to be hypoxic regions. These results indicate that Dlx-2 expression is closely related to microenvironmental stresses, such as hypoxia and GD.

### Dlx-2 expression in solid human tumors

**Figure 3:**
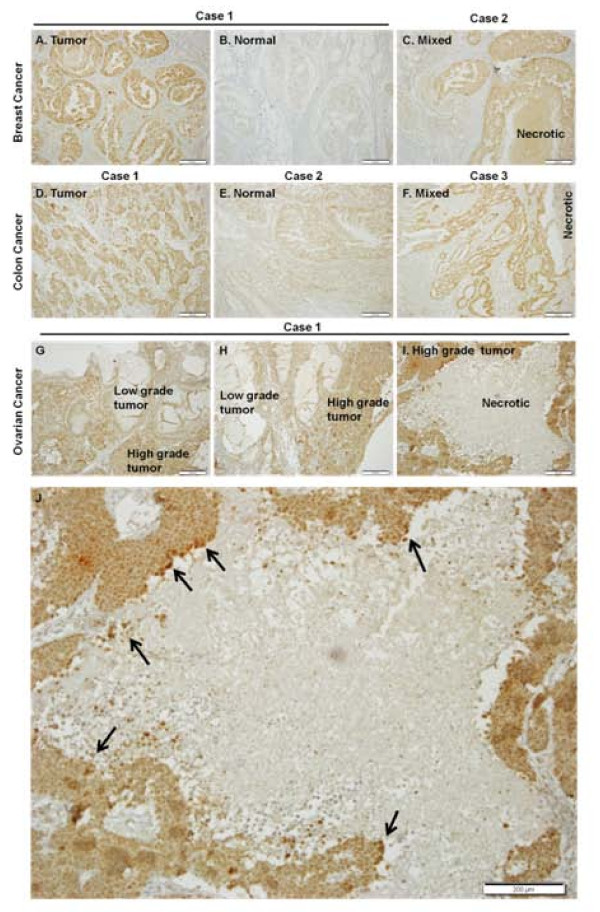
**Immunohistochemical detection of Dlx-2 in human tumors, including breast, colon, and ovarian cancers**. IHC was performed on 4-μm sections of formalin-fixed, paraffin-embedded human tumors, including breast, colon, and ovarian tumor tissues. Sections were incubated with an anti-Dlx-2 antibody and the antibody was visualized with diaminobenzidine chromogen, and sections were counterstained with hematoxylin. Dlx-2, brown staining; nuclei, blue staining (H & E). A-C, breast cancer; D-F, colon cancer; G-I, ovarian cancer, and J, the enlargement image of panel I. Arrows in panel J indicate strong positive Dlx-2 staining in tumor cells adjacent to areas of necrosis and in the cells in the necrotic core. Scale bar, 200 μm.

To study Dlx-2 expression in human tumors, we performed real-time PCR using the RNAs extracted from paired biopsy breast, colon, and ovarian cancer tissues and the corresponding normal tissues. Dlx-2 expression was higher in breast and ovarian cancer tissues compared with adjacent normal tissues (Additional file 2. Figure S2). In colon cancer tissues, Dlx-2 expression varied; some colon cancer tissues had high levels of Dlx-2 mRNA, whereas others had Dlx-2 mRNA levels similar to or lower than those observed in normal tissue. We also assessed the expression of Dlx-2 protein in human tumors, including breast, colon, and ovarian cancers, with immunohistochemical staining (IHC) in paraffin-embedded and formalin-fixed tissues and compared the results with those of real-time PCR analysis (Figure 3). Immunoreactivity for Dlx-2 in the tumor cells was found exclusively in the nucleus. In breast and colon cancers, Dlx-2 expression higher in tumor tissues than in matched non-tumorigenic tissues and stromal cells around cancer cells, i.e., fibroblasts and lymphocytes (Figure 3A-F). Furthermore, in ovarian cancer, Dlx-2 expression was detected in high-grade tumors that were poorly differentiated but not in the low-grade tumors, which were well differentiated (Figure 3G and 3H). Thus, Dlx-2 expression was related to poor differentiation grade of tumor, indicating a role(s) of Dlx-2 in tumor development. It is noteworthy that strong positive Dlx-2 staining was observed in tumor cells adjacent to areas of necrosis and in the cells in the necrotic core (Figure 3I and 3J). These results further support that Dlx-2 expression is related to metabolic stresses such as hypoxia and GD. However, because Dlx-2 was also expressed throughout tumor tissues, its expression is likely to be regulated also by stimuli other than metabolic stress and plays an important role(s) in tumor development.

### Dlx-2 shRNA prevents metabolic stress-induced necrosis in two-dimensional cell culture

**Figure 4:**
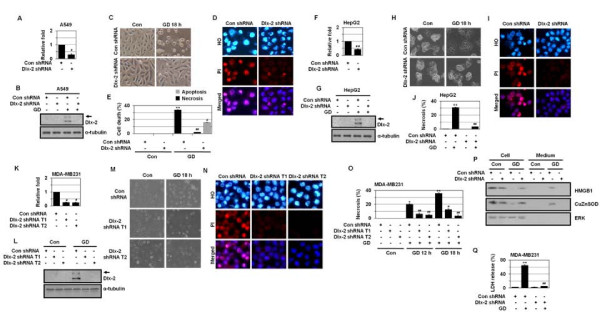
**Dlx-2 plays a role(s) in metabolic stress-induced necrosis**. (A-E) A549 cells were stably transfected with Dlx-2 shRNA. The cells were analyzed by real-time PCR for Dlx-2 expression (A), and treated with GD 12 h and analyzed using Western blotting (B). The cells were treated with GD for 18 h (C) and stained with HO/PI (D), and apoptotic and necrotic cells were scored (E). (F-J) HepG2 cells were stably transfected with Dlx-2 shRNA. The cells were analyzed by real-time PCR for Dlx-2 expression (F), and treated with GD for 12 h and analyzed using Western blotting (G). The cells were treated with GD for 18 h (H), stained with HO/PI (I), and apoptotic and necrotic cells were scored (J). (K-Q) MDA-MB-231 cells were stably transfected with 2 different Dlx-2 shRNA (T1 and T2). The cells were analyzed by real-time PCR for Dlx-2 expression (K), and treated with GD for 12 h and analyzed using Western blotting (L). The cells were treated with GD for 18 h (M), stained with HO/PI (N), and apoptotic and necrotic cells were scored (O). The cells were treated with GD for 12 h and analyzed for HMGB1 (P) and LDH release (Q). The values obtained from real-time PCR, HO/PI staining, and LDH release assay are expressed as mean ± SE (n = 3). *P < 0.05, **P < 0.01 versus control; ^#^*P *< 0.05, ^##^*P *< 0.01 versus control shRNA. Arrow in panels B, G, and L, a putative modified form of Dlx-2.

We investigated whether Dlx-2 is functionally linked to GD-induced necrosis using specific transcript knockdown with short hairpin RNA (shRNA). We used 2 different shRNA oligonucleotides: one (target 1) was a 19-mer shRNA oligonucleotide directed to the N-terminal region (position from 637 to 655) of human Dlx-2 mRNA sequence [Accession No. NM_004405 GenBank: BC032558.1] and another (target 2) was a 25-mer shRNA oligonucleotide directed to the 3' UTR region (position from 1231 to 1255) of human Dlx-2 mRNA sequence [Accession No. NM_004405 GenBank: BC032558.1]. These 2 oligonucleotides were not directed to other human Dlx mRNA. Dlx-2 shRNA was verified to be effective in knocking down Dlx-2 mRNA levels in A549, HepG2, and MDA-MB-231 cell lines, as determined by real-time PCR (Figure 4A, F, and 4K). Dlx-2 shRNA also prevented the GD induction of Dlx-2, as determined by Western blotting (Figure 4B, G, and 4L), thereby indicating that Dlx-2 shRNA specifically suppresses the expression and function of Dlx-2.

We examined the effects of Dlx-2 shRNA on GD-induced necrosis. Dlx-2 shRNA significantly inhibited metabolic stress-induced cell rounding (Figure 4C, H, and 4M) and increase in cell populations that had intact pink nuclei in Hoechst 33342 (HO)/propidium iodide (PI) staining in A549, HepG2, and MDA-MB-231 cells (Figure 4D, E, I, J, N, and 4O). In A549 cells, while Dlx-2 shRNA repressed the GD-induced increase in population of PI-positive cells, it increased cells with condensed/fragmented blue nuclei (HO/PI double staining) (Figure 4D and 4E), indicating that Dlx-2 shRNA switches GD-induced necrotic cell death to apoptosis. In contrast, when GD-induced necrosis was inhibited by Dlx-2 shRNA in HepG2 and MDA-MB-231 cells, apoptosis did not occur as an alternative death mechanism (Figure 4I, J, N, and 4O). We also observed that Dlx-2 shRNA suppressed the GD-induced release of HMGB1 into the extracellular space (Figure 4P and Additional file 3. Figure S3). Dlx-2 shRNA transfection also prevented necrosis-linked lactate dehydrogenase (LDH) release (Figure 4Q). These results indicate that Dlx-2 is implicated in metabolic stress-induced necrosis.

### Dlx-2 shRNA prevents metabolic stress-induced necrosis in MTSs

**Figure 5:**
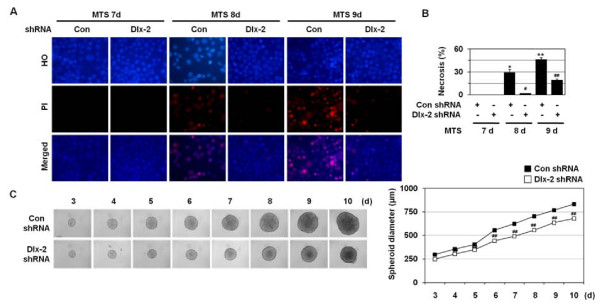
**Dlx-2 shRNA prevents metabolic stress-induced necrosis in MTS**. (A, B) MCF-7 cells stably transfected with control and Dlx-2 shRNA were seeded into 1.2% agarose-coated 96-well plates at a density of 400 cells per well and cultured for 7, 8, and 9 days. Then the cells were dissociated and stained with HO/PI (A), and apoptotic and necrotic cells were scored. The values are expressed as mean ± SE (n = 3). *P < 0.05, **P < 0.01 versus control; ^#^*P *< 0.05; ^##^*P *< 0.01 versus control shRNA (B). (C) Formation, growth, and morphology of MTSs made using MCF-7 control and Dlx-2 shRNA stable cells. To calculate MTS size, diameters of 5 spheroids were measured every day. Results are expressed as mean ± SE (n = 3). ^##^*P *< 0.01 versus control shRNA.

We examined the effects of Dlx-2 shRNA on necrosis, using MTSs. As demonstrated previously [33,39], PI-positive cells were detected in 8-9 day MTSs but not in 7-day MTSs (Figure 5A). Dlx-2 shRNA prevented necrosis, as revealed by a prominent reduction in the population of cells that had pink nuclei with HO/PI staining at 8-9 days in MCF-7 MTS culture (Figure 5A and 5B). We also observed that stable Dlx-2 silencing in MCF-7 MTSs slightly suppressed the growth of the MCF-7-day MTSs (Figure 5C).

### Dlx-2 shRNA prevents metabolic stress-induced mitochondrial ROS production, loss of mitochondrial membrane potential, and mitochondrial permeability transition

**Figure 6:**
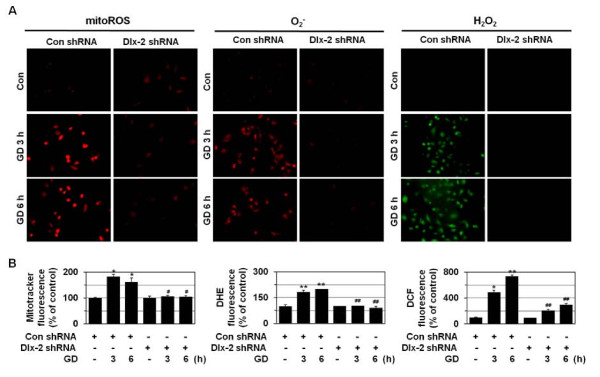
**Dlx-2 shRNA prevents metabolic stress-induced ROS production**. **(A, B) **MDA-MB-231 cells were stably transfected with control or Dlx-2 shRNA and treated with GD for 3 or 6 h, and mitochondrial ROS and O_2_^- ^and intracellular H_2_O_2 _production was measured using the MitoTracker Red CM-H_2_XRos, DHE, and DCFH-DA, respectively, under a fluorescence microscope (X200; Carl Zeiss). Representative images of cells from 3 independent experiments are shown (A). The values are expressed as mean ± SE from approximately 200 cells per treatment group (n = 3) (B). *P < 0.05, **P < 0.01 versus control; ^#^*P *< 0.05; ^##^*P *< 0.01 versus control shRNA.

Mitochondrial O_2_^- ^is produced especially at Complex I or Complex III of the electron transport chain [41,42], and its levels increase upon GD treatment to mediate GD-induced cytotoxicity and cell death [34-36]. As shown in Figure 6, GD significantly enhances the production of mitochondrial ROS, O_2_^- ^and intracellular H_2_O_2_, as revealed by staining with 3 different fluorogenic probes: MitoTracker Red CM-H_2_XRos, dihydroethidium (HE), and 2¢,7¢-dichlorofluorescein diacetate (DCFH-DA). Dlx-2 interference blocked the GD-induced production of mitochondrial ROS, O_2_^- ^and intracellular H_2_O_2 _(Figure 6), indicating that Dlx-2-mediated necrosis regulation is linked to its ability to control metabolic stress-induced ROS production.

**Figure 7:**
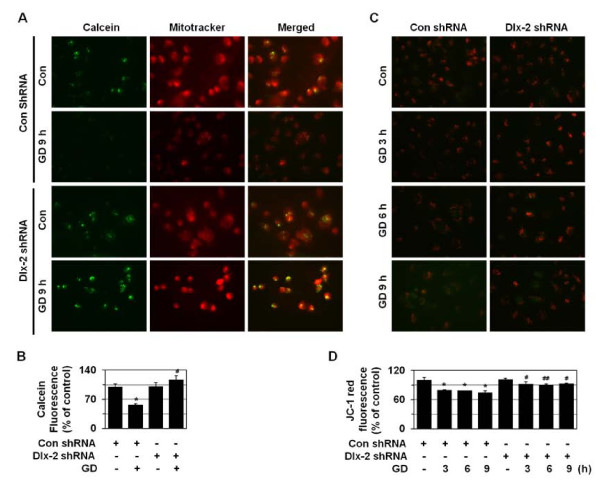
**Dlx-2 shRNA prevents metabolic stress-induced loss of ΔΨm and mPT**. (A, B) MDA-MB-231 cells were stably transfected with control or Dlx-2 shRNA and treated with GD for 9 h and loaded with 0.5 μM calcein AM and 5 mM CoCl_2 _for the final 15 min of the incubation. To detect cytoplasmic mitochondrial distribution, 50 nM MitoTracker CMX-ROS were added during calcein loading. Calcein fluorescence was excited at 488 nm and emitted at 515 nm; MitoTracker Red CMX-ROS was excited at 579 nm, and emitted at 599 nm; and the cells were observed using fluorescence microscopy. Representative images of cells from 3 independent experiments were shown (A). The results are expressed as mean ± SE from 15 to 30 cells per treatment group (n = 3) (B). *P < 0.05 versus control; ^#^*P *< 0.01 versus control shRNA. (C, D) MDA-MB-231 cells stably transfected with control or Dlx-2 shRNA were treated with GD for the indicated times and then treated with 5 mg/ml JC-1 for 15 min. Representative images of cells from 3 independent experiments are shown (C). The results are expressed as mean ± SE from 50 to 100 cells per group (n = 3) (D). *P < 0.05 versus control; ^#^*P *< 0.05; ^##^*P *< 0.01 versus control shRNA.

ROS are known to be able to induce the mitochondrial permeability transition (mPT) pore opening in the mitochondrial inner membrane, while the transient mPT pore opening induces apoptosis, and its prolonged opening results in necrosis [43]. The mitochondrial membrane potential (ΔΨm) is also lost upon the mPT pore opening. If the mPT pore is open for longer periods, cells cannot generate ATP by oxidative phosphorylation, leading to necrotic cell death as a consequence of ATP depletion. Thus, we examined mPT by cobalt-quenched calcein (CoQC) measurement. We observed that calcein fluorescence was lost following the opening of the mPT pore upon GD treatment, and Dlx-2 shRNA prevented this mPT pore opening (Figure 7A and 7B). We also measured the ΔΨm of cells with JC-1, a mitochondria-specific and lipophilic-cationic fluorescence dye. While red J-aggregate fluorescence appeared to be progressively lost upon GD treatment and cytoplasmic diffusion of green monomer fluorescence was detected, Dlx-2 shRNA inhibited this GD-induced decline in ΔΨm (Figure 7C and 7D). Thus, Dlx-2 shRNA is likely to inhibit metabolic stress-induced necrosis by preventing mitochondrial ROS production and subsequent loss of mitochondrial membrane potential and mitochondrial permeability transition, which are the primary events that trigger necrosis.

## Discussion

### Dlx-2 is implicated in necrosis

A growing number of homeobox genes are deregulated in a variety of human tumors [20-23]. Deregulation of the expression of Dlx genes, including Dlx-4 and Dlx-5, was found in human solid tumors and hematologic malignancies and indicates an important role(s) of Dlx in tumor growth and progression [24-30]. Here, we show that Dlx-2 is induced in cancer cells that die by necrosis in response to metabolic stress (Figure 1). Increased Dlx-2 expression was also detected in the inner regions, which experience metabolic stress, of human tumors (Figure 3) and of MTSs (Figure 2). We further found that Dlx-2 shRNA inhibited metabolic stress-induced increase in propidium iodide-positive cell populations and HMGB1 and LDH release, indicating a critical role(s) of Dlx-2 in metabolic stress-induced necrosis (Figure 4). In A549 cells, Dlx-2 shRNA repressed GD-induced increase in the population of PI-positive cells but increased the number of cells with condensed/fragmented blue nuclei (HO/PI double staining) (Figure 4D and 4E), thus indicating that Dlx-2 shRNA switches GD-induced necrotic cell death to apoptosis. In the case of HepG2 and MDA-MB-231 cells, Dlx-2 shRNA prevented necrosis but without increasing the number of apoptotic cells (Figure 4I, J, N, and 4O). Previously, we showed that pretreatment of the anti-oxidant NAC switched GD-induced necrosis to apoptosis in A549 cells, whereas it switched it to autophagy-like cell death in HepG2 and MDA-MB-231 cells [44]. Thus, Dlx-2 shRNA-transfected cells can undergo either apoptosis or other cell fates (including autophagic cell death) upon GD, depending on the cell types due to their different cellular context.

Although Dlx-2 shRNA inhibited metabolic stress-induced necrosis, Dlx-2 overexpression did not trigger necrosis, although it caused an alteration in cell morphology to the mesenchymal cell-like phenotype (Additional file 1. Figure S1C), indicating that Dlx-2 is necessary but not sufficient for metabolic stress-induced necrosis. In agreement with this observation, Dlx-2 was expressed throughout tumor tissues (Figure 3), indicating that its expression is also regulated by stimuli other than metabolic stress. Necrosis is accompanied by several different processes, including mitochondrial dysfunction, excess ROS production, and ATP depletion [6,7]. Thus, Dlx-2 may trigger necrosis if tumor cells are under such a metabolic stress environment. In other words, in the absence of metabolic stress, Dlx-2 may promote tumor growth and progression by unknown mechanisms, but in the presence of metabolic stress, it may facilitate metabolic stress-induced necrosis by promoting mitochondrial ROS production. Our results suggest that Dlx-2 may be implicated in tumor progression via the regulation of metabolic stress-induced necrosis.

### Regulation of cellular redox status by Dlx-2

Our results showed that GD-induced expression of Dlx-2 is ROS-dependent (Figure 1), and GD-induced production of ROS is also Dlx-2-dependent (Figure 6). ROS produced under stress conditions are known to spread from one mitochondrion to neighboring mitochondria in a process known as ROS-induced ROS release (RIRR) for enhanced ROS production, [45,46]. Dlx-2 induced upon metabolic stress may facilitate ROS production, which in turn enhance Dlx-2 expression to accelerate massive ROS production by RIRR and induce GD-induced cytotoxicity and necrosis, thereby constituting a positive feedback mechanism between Dlx-2 expression and cellular ROS levels. Mitochondrial ROS massively produced through RIRR may oxidize HMGB1 released by necrosis and the oxidized HMGB1 may exert its activity to trigger apoptosis. However, because many proapoptotic molecules such as p53 and caspases are aggregated to an inactive form upon GD treatment [44], GD is likely to induce necrosis instead of apoptosis.

How does Dlx-2 control mitochondrial ROS production in response to GD? Mitochondrial O_2_^- ^is produced even under normal conditions [41,42], and its production is enhanced by GD treatment and triggers necrotic cell death. Mitochondrial dysfunction has been linked to the induction of necrosis. Tumor cells have been shown to have abnormal mitochondrial structure and DNA integrity [47,48], and these mitochondrial deregulations have been suggested to make tumor cells more sensitive to oxidative stress and cell killing induced by GD and 2-deoxyglucose, a glycolysis inhibitor [36]. In addition, tumor cells with dysregulated mitochondria undergo necrosis instead of apoptosis in response to alkylating DNA damage that induces rapid ATP depletion through PARP activation [49]. We speculate that Dlx-2 may affect mitochondrial function and sensitize tumor cells to metabolic stress and death by necrosis. HMGB1 is known to enhance the activities of a number of transcription factors, including p53 and the Rel/NF-κB family. Our preliminary data showed that HMGB1 shRNA prevents metabolic stress-induced necrosis (data not shown). These results indicate that HMGB1 may be implicated in Dlx-2-mediated necrosis. We are investigating if Dlx-2 regulates metabolic stress-induced necrosis by affecting mitochondrial function and if HMGB1 plays a role(s) in Dlx-2-mediated necrosis.

## Conclusion

Necrosis promotes tumor progression and aggressiveness. Consequently, necrosis has important clinical implications in tumor development, but its molecular mechanism has been less investigated. In this study, we show that Dlx-2, a homeobox gene of the Dlx family that is involved in embryonic development, is implicated in metabolic stress-induced necrosis via the regulation of metabolic stress-induced increases in mitochondrial ROS, which are responsible for triggering necrosis. These results suggest that Dlx-2 may be implicated in tumor progression via the regulation of metabolic stress-induced necrosis.

## Materials and methods

### Cell culture, chemical treatment, and MTS culture

A549, MDA-MB-231, HepG2, HCT116, and HeLa cells were obtained from American Type Culture Collection, maintained in RPMI-1640 or DMEM supplemented with 10% (v/v) heat-inactivated fetal bovine serum (HyClone, Logan, UT) and 1% penicillin-streptomycin (HyClone, Logan, UT) in a 37°C humidified incubator with 5% CO_2_, and treated with GD [32]. To induce GD-induced necrosis-to-apoptotis switch in A549 cells, the cells were pre-treated with 100 nM PMA for 30 min and treated with GD. NAC (10 mM) was pretreated for 1 h and treated with GD. H_2_O_2 _(300 μM) and menadione (10 μM) were treated to cells for 48 h. For the MTS culture, MCF-7 cells (provided by Dr. JI Yook, University of Yonsei, Korea) were seeded at a density of 400 cells in 200 μl medium into 1.2% agarose-precoated 96-well plates. After 3 days of culture, 100 μl of medium was replaced with fresh medium every 2 days. To determine the MTS growth, the diameters of spheroids were measured every day. For analysis of cell death patterns, MTSs were trypsinized and then stained with HO/PI as described below. To determine the expression of Dlx-2 within MTSs, MTSs were dissociated into subpopulations of cells from 4 different locations in the spheroid as described by LaRue *et al. *[40]. The cells isolated from different locations within spheroids were analyzed by Western blotting, reverse transcription-polymerase chain reaction (RT-PCR), and real-time PCR as described below.

### Microarray

Microarrays were performed to screen for the differentially expressed genes using Operon Human Whole 35 K Oligo chips (GenoCheck, Korea); a complete listing of the genes on this microarray is available at the web site: http://www.genocheck.com. Data analysis was carried out using GeneSpring GX 7.3 (Agilent Technologies), and the values were normalized using the LOWESS algorithm. The Affymetrix microarray data have been deposited in the Gene Expression Omnibus (GEO) database (GEO accession no. GSE24271).

### Western blotting, HMGB1 release assay, LDH release assay, RT-PCR, and real-time PCR

Western blotting analyses were performed as described previously using the following antibodies: Dlx-2 (Chemicon, France), α-tubulin (Biogenex, CA), HMGB1 (BD Pharmingen, CA), CuZnSOD, (Santa Cruz, CA), ERK1/2 (Cell Signaling, MA) [32]. A HMGB1 release assay was carried out as described previously [32]. LDH release was measured using the LDH Cytotoxicity Detection Kit (Roche Applied Science) according to the manufacturer's instructions. Transcript levels were assessed with RT-PCR and quantitative real-time PCR with primers for Dlx-2 and β-actin (Additional file 4. Table S1).

### HO/PI staining, immunofluorescence, and confocal microscopy

GD-induced cell death mode was determined by HO/PI double staining as described previously [32]. HO crosses the plasma membrane of all cells that are viable or damaged, resulting in blue fluorescence, whereas PI only penetrates cells with damaged membranes, leading to nuclear red fluorescence. Thus, intact blue nuclei, condensed/fragmented blue nuclei, condensed/fragmented pink nuclei, and intact pink nuclei were considered to indicate viable, early apoptotic, late apoptotic (secondary necrotic), and necrotic cells, respectively. Apoptotic and necrotic cells were scored using 500 to 800 cells per group (n = 3). Intracellular H_2_O_2_, O_2_^-^, and mitochondrial ROS were detected using the DCFH-DA (Molecular Probes; 50 μM), HE (Molecular Probes; 10 μM), and MitoTracker Red CM-H_2_XRos (Molecular Probes, 50 nM), respectively, by fluorescence microscopy. Next, mPT pore opening and ΔΨm were analyzed using CoQC and JC-1 staining, respectively, as described previously [33]. Fluorescence intensity was analyzed with Axiovision LE software (Release 4.8 version).

### Dlx-2 transfection and shRNA interference

pCAGGS-Dlx-2 (provided by Dr. John L.R. Rubenstein, University of California at San Francisco) was constructed by inserting the Dlx-2 open reading frame into pCAGGS (BCCM/LMPD, Belgium). The vectors pCAGGS and pCAGGS-Dlx-2 were transfected into MCF-7 using jetPEI (Polyplus transfection) according to manufacturer's protocol. pSUPER-Dlx-2 shRNA was generated from 2 different annealed oligonucleotides (target 1, 5'-GATCCCCTTCGGATAGTGAACGGGAATTCAAGAGATTCCCGTTCACTATCCGAATTTTTA-3' and 5'-AGCTTAAAAATTCGGATAGTGAACGGGAATCTCTTGAATTCCCGTTCACTATCCGAAGGG-3' and target 2, 5'-GATCCCCAGAGACCACTTATCCTCATTGCTTATTCAAGAGATAAGCAATGAGGATAAGTGGTCTCT

TTTTTA- 3' and 5' AGCTTAAAAAAGAGACCACTTATCCTCATTGCTTATCTCTTGAATAAGCAATGAGGATAAGTGGTC

TCTGGG-3') [Accession No.  GenBank: ] that were inserted into the *Hin*dIII and *Bgl*II sites of pSUPER.gfp/neo (Oligoengine Platform, Seattle, WA); the human Dlx-2 target sequence is underlined. Control shRNA was generated from annealed oligonucleotides (5'-GATCCCCAATTCTCCGAACGTGTCACGTTTCAAGAGAACGTGACACGTTCGGAGAATTTTTTTA-3' and

5'-AGCTTAAAAAAATTCTCCGAACGTGTCACGTTCTCTTGAAACGTGACACGTTCGGAGAATTGGG-3') inserted into the *Hin*dIII and *Bgl*II sites of pSUPER.gfp/neo (Oligoengine Platform, Seattle, WA). All target sequences were designed and verified as specific for Dlx-2 by BLAST searching against the human genome and real-time PCR, respectively. The vectors pSUPER-control and pSUPER-Dlx-2 shRNA were transfected using jetPEI, and stable cell line selection performed with 1-2 mg/ml G418. Several clones were isolated after shRNA transfection in each cell type and individually characterized.

### Real-time analysis and immunohistochemical staining for Dlx-2 expression in human cancer issues

The expression of the Dlx-2 gene in normal and cancerous human tissues was estimated by real-time PCR. Frozen cancer and normal matched tissue pairs from the same individuals were provided by the National Biobank of Korea, PNUH. For RNA extraction, tissues were added to 1 ml of Trizol reagent (Invitrogen, NY) and vortexed twice for 10 s each time, using the FastPrep-24 system (MP Biomedicals LLC.). After vortexing, tissue lysates were quick chilled on ice, and then the procedure was continued in accordance with the manufacturer's protocol. Isolated 2 μg of total RNA were used as the template, and reverse transcription was performed in the presence of M-MLV reverse transcriptase (Invitrogen), 5× first strand buffer, RNase inhibitor, oligo (dT)20, dNTP, and DTT, according to manufacturer's protocol using Thermal Block (MyGenie96, Bioneer, Korea).

IHC was performed on 4-μm sections of formalin-fixed, paraffin-embedded human cancer tissues (Department of Pathology, College of Medicine, Chosun University). Sections were deparaffinized in xylene and graded alcohol. Antigen retrieval was performed by autoclaving for 15 min. After incubation with blocking solution for 30 min, sections were incubated with anti-Dlx-2 antibody for 1 h, biotinylated secondary antibody for 20 min, and then with streptavidin horseradish peroxidase for 10 min. Staining was carried out with diaminobenzidine chromogen and counterstaining with hematoxylin.

### Statistical analysis

All experiments were independently performed at least 3 times. Data were analyzed by the Student's *t*-test and *P *< 0.05 was considered statistically significant.

## Abbreviations

CoQC: cobalt-quenched calcein; CuZnSOD: copper-zinc superoxide dismutase; Dll: Drosophila distal-less; Dlx: Distal-less; GD: glucose deprivation; HMGB1: high mobility group box 1; IHC: immunohistochemistry; IL-1a: interleukin-1a; ROS: reactive oxygen species; ΔΨm: mitochondrial membrane potential; MnSOD: manganese superoxide dismutase; mPT: mitochondrial permeability transition; MTSs: multicellular tumor spheroids; NAC: *N*-acetylcysteine; PMA: phorbol-12-myristate-13-acetate; RAGEs: receptor for advanced glycation end products; RIRR: ROS-induced ROS release; shRNA: short hairpin RNA; TLR: Toll-like receptor.

## Competing interests

The authors declare that they have no competing interests.

## Authors' contributions

SYL performed most of the experiments and participated in designing the study, analyzing the data, and drafting the manuscript. HMJ designed and performed the experiments and participated in analyzing the data and drafting the manuscript, MKJ, CHK, and HSB performed the experiments, HGP and S-CL analyzed the data, SIH performed the experiments and analyzed the data and drafting the manuscript. HSK conceptualized the study, participated in its design, and helped analyze the data and draft the manuscript.

All authors read and approved the final manuscript.

## Supplementary Material

Additional file 1 **Figure S1. Full scan blots**. (A) Full scan of blot depicted in Figure 1D. A549 cells were pretreated with PMA (100 nM) for 30 min and treated with GD for 12 h. The cells were analyzed using Western blotting with antibodies against Dlx-2 and α-tubulin (10 μg protein extract). (B) Full scan of blot depicted in Figure 1F. A549, HepG2, MDA-MB-231, HCT116, and HeLa cells were treated with GD for the indicated times and then analyzed using Western blotting with antibodies against Dlx-2 and α-tubulin (10 μg protein extract). (C) MCF-7 cells were transiently transfected with a control or Dlx-2 expression vector for 2 d and cell morphology was examined using phase-contrast microscopy and photographed under a magnification of 400×. Dlx-2 expression was analyzed using Western blotting with antibodies against Dlx-2 and α-tubulin. Arrow, a putative modified form of Dlx-2. Click here for file

Additional file 2 **Figure S2. Real-time PCR analysis for expression of Dlx-2 in human tumors, including breast, colon, and ovarian cancers**. Dlx-2 expression was analyzed with real-time PCR using the RNAs extracted from paired biopsy breast, colon, and ovarian cancer tissues and the corresponding normal tissues. Values are normalized to β-actin. *P < 0.05, **P < 0.01 versus normal tissues. N, normal tissues; T, tumors. Click here for file

Additional file 3 **Figure S3. Ponceau S staining pattern of Figure **4P. MDA-MB-231 cells that were stably transfected with control or Dlx-2 shRNA were treated with GD for 12 h, and both medium and cell pellets were prepared and analyzed with SDS-PAGE and Ponceau S staining. Click here for file

Additional file 4 **Table S1. Primer sequences for RT-PCR and real time PCR**. Click here for file
