# Supplementary figures and images for: Homeobox gene Dlx-2 is implicated in metabolic stress-induced necrosis
Source: Mol Cancer. 2011 Sep 14;10:113. doi: 10.1186/1476-4598-10-113 (PMC3181206; doi:10.1186/1476-4598-10-113)

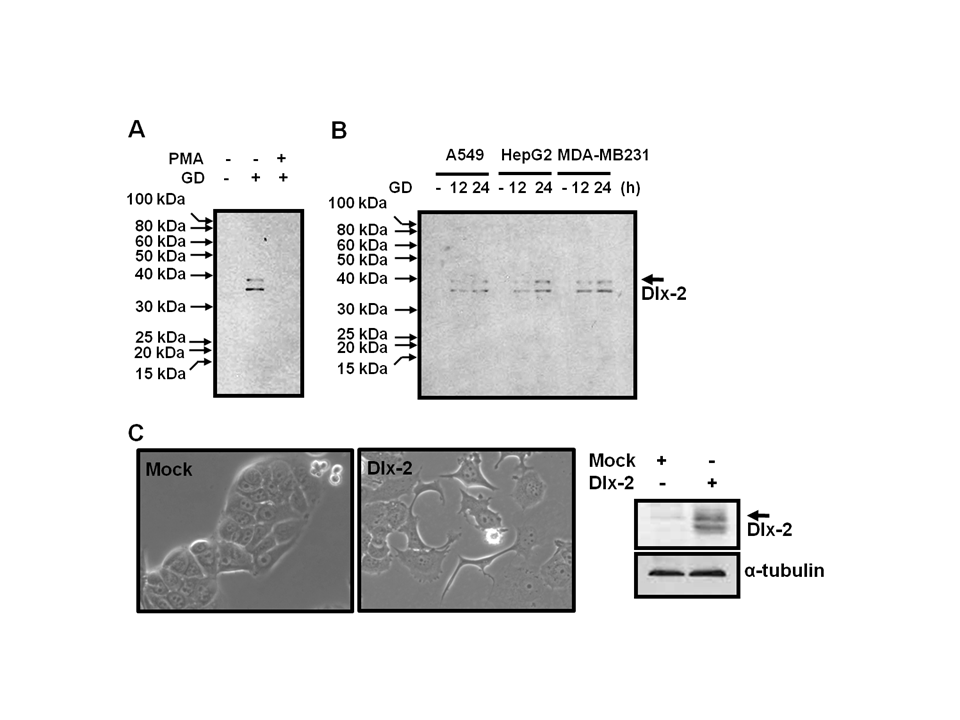

Supplement: Additional file 1 — Figure S1. Full scan blots. (A) Full scan of blot depicted in Figure 1D. A549 cells were pretreated with PMA (100 nM) for 30 min and treated with GD for 12 h. The cells were analyzed using Western blotting with antibodies against Dlx-2 and α-tubulin (10 μg protein extract). (B) Full scan of blot depicted in Figure 1F. A549, HepG2, MDA-MB-231, HCT116, and HeLa cells were treated with GD for the indicated times and then analyzed using Western blotting with antibodies against Dlx-2 and α-tubulin (10 μg protein extract). (C) MCF-7 cells were transiently transfected with a control or Dlx-2 expression vector for 2 d and cell morphology was examined using phase-contrast microscopy and photographed under a magnification of 400×. Dlx-2 expression was analyzed using Western blotting with antibodies against Dlx-2 and α-tubulin. Arrow, a putative modified form of Dlx-2. [file 1476-4598-10-113-S1.TIFF]

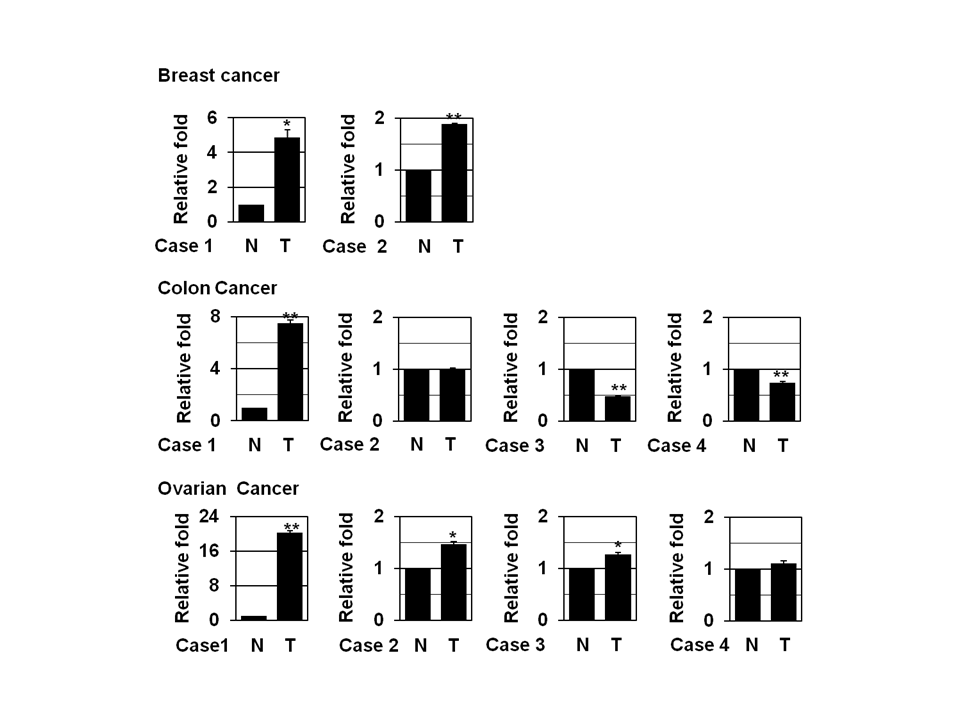

Supplement: Additional file 2 — Figure S2. Real-time PCR analysis for expression of Dlx-2 in human tumors, including breast, colon, and ovarian cancers. Dlx-2 expression was analyzed with real-time PCR using the RNAs extracted from paired biopsy breast, colon, and ovarian cancer tissues and the corresponding normal tissues. Values are normalized to β-actin. *P < 0.05, **P < 0.01 versus normal tissues. N, normal tissues; T, tumors. [file 1476-4598-10-113-S2.TIFF]

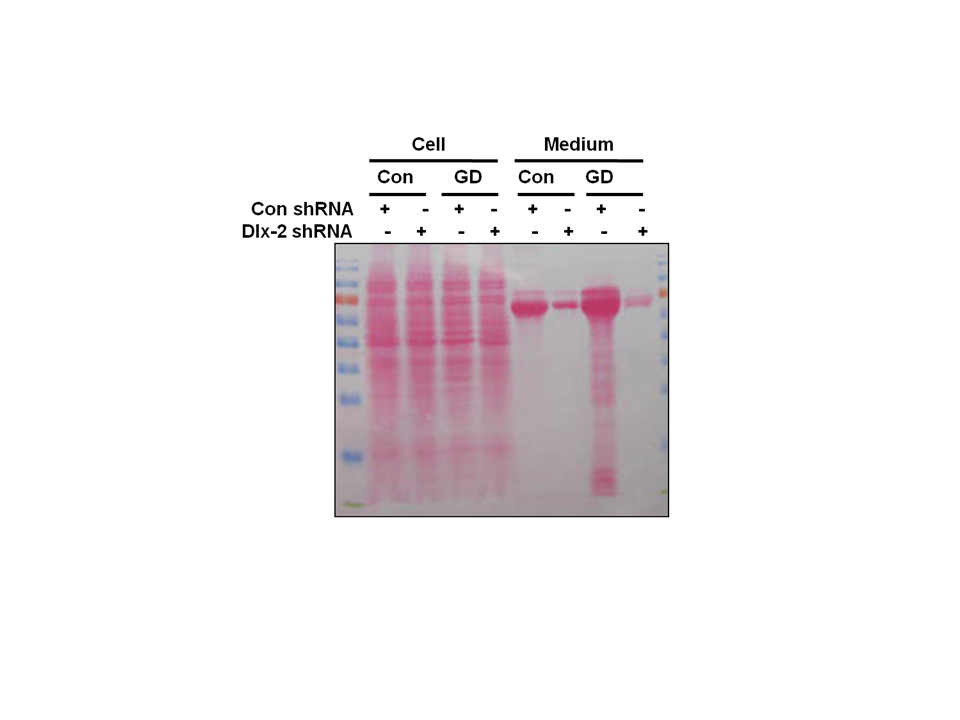

Supplement: Additional file 3 — Figure S3. Ponceau S staining pattern of Figure 4P. MDA-MB-231 cells that were stably transfected with control or Dlx-2 shRNA were treated with GD for 12 h, and both medium and cell pellets were prepared and analyzed with SDS-PAGE and Ponceau S staining. [file 1476-4598-10-113-S3.TIFF]

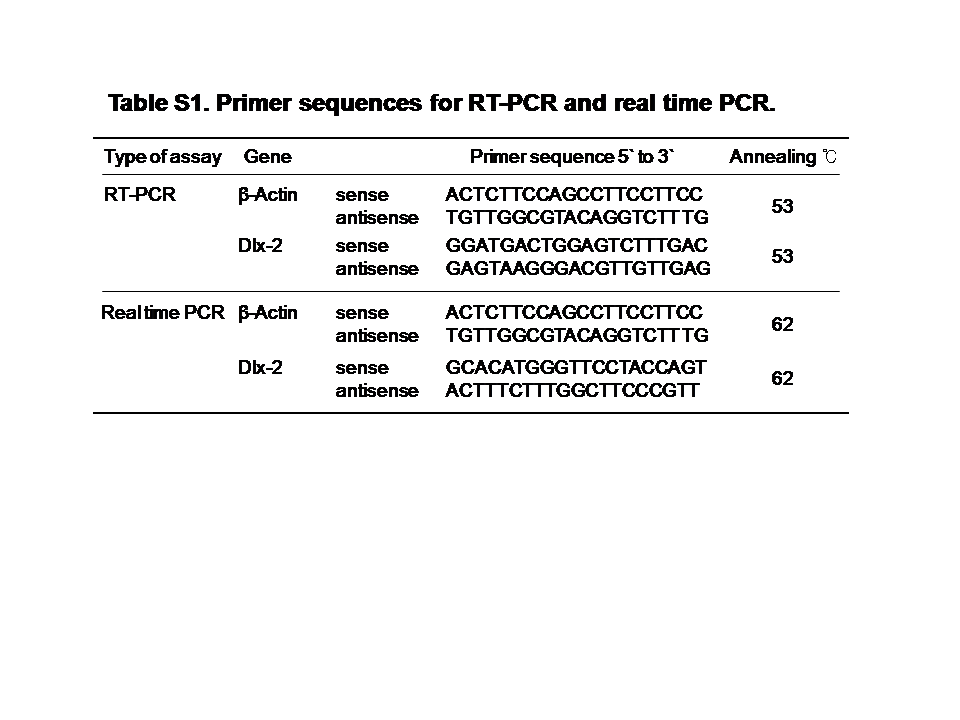

Supplement: Additional file 4 — Table S1. Primer sequences for RT-PCR and real time PCR. [file 1476-4598-10-113-S4.TIFF]
